# Percutaneous retrieval of the lattice-tip catheter entrapped in post-surgical atrioventricular valve apparatus: two case reports

**DOI:** 10.1093/ehjcr/ytag077

**Published:** 2026-02-13

**Authors:** Nicoletta Ventrella, Petr Peichl, Corrado Carbucicchio, Predrag Stojadinović, Marco Schiavone, Kazuto Hayasaka, Claudio Tondo, Josef Kautzner

**Affiliations:** Department of Electrophysiology, Institute for Clinical and Experimental Medicine (IKEM), Vídeňská 1958, 140 21 Praha 4, Prague, Czechia; Second Faculty of Medicine, Charles University, Plzeňská 311, 150 06 Praha 5-Motol, Prague, Czechia; Department of Clinical Electrophysiology and Cardiac Pacing, Centro Cardiologico Monzino, IRCCS, Via Privata Carlo Parea, 4, 20138 Milano MI, Italy; Department of Electrophysiology, Institute for Clinical and Experimental Medicine (IKEM), Vídeňská 1958, 140 21 Praha 4, Prague, Czechia; Department of Clinical Electrophysiology and Cardiac Pacing, Centro Cardiologico Monzino, IRCCS, Via Privata Carlo Parea, 4, 20138 Milano MI, Italy; Department of Electrophysiology, Institute for Clinical and Experimental Medicine (IKEM), Vídeňská 1958, 140 21 Praha 4, Prague, Czechia; Department of Clinical Electrophysiology and Cardiac Pacing, Centro Cardiologico Monzino, IRCCS, Via Privata Carlo Parea, 4, 20138 Milano MI, Italy; Department of Electrophysiology, Institute for Clinical and Experimental Medicine (IKEM), Vídeňská 1958, 140 21 Praha 4, Prague, Czechia; Department of Clinical Electrophysiology and Cardiac Pacing, Centro Cardiologico Monzino, IRCCS, Via Privata Carlo Parea, 4, 20138 Milano MI, Italy; Department of Biomedical, Surgical and Dental Sciences, Università degli Studi di Milano, Via Festa del Perdono, 7, 20122 Milano MI, Italy; Department of Electrophysiology, Institute for Clinical and Experimental Medicine (IKEM), Vídeňská 1958, 140 21 Praha 4, Prague, Czechia

**Keywords:** Entrapment, Lattice-tip catheter, Large footprint catheter, Ventricular tachycardia ablation, Retrograde, Transaortic, Case report

## Abstract

**Background:**

The novel lattice-tip catheter (Sphere-9, Medtronic) enables high-resolution mapping and the ability to switch between radiofrequency and pulsed electric field. Initial experience has shown promising results for treating complex ventricular tachycardias (VTs). However, safety data remain limited, particularly regarding catheter–valve interactions.

**Cases summary:**

We report two cases of mechanical entrapment of the lattice-tip catheter in the atrioventricular valves during VT ablation performed via a retrograde transaortic approach. In both cases, the catheter became entangled within the subvalvular apparatus—specifically, in the tricuspid valve of a patient with congenitally corrected transposition of the great arteries and in the mitral valve of a patient who had previously undergone mitral repair. Standard retrieval manoeuvres were unsuccessful, and resolution required percutaneous bailout strategies, specifically transseptal access with bioptomes or forceps. Both procedures were completed without long-term sequelae. These are the first reported cases of large-footprint catheter entrapment in the atrioventricular valve apparatus, highlighting potential mechanical risks associated with its employment in the ventricular setting.

**Discussion:**

Our experience emphasizes the importance of preferring transseptal access over retrograde access where feasible, due to its more favourable trajectory and the ability to support safer rescue techniques. Real-time imaging, particularly intracardiac echocardiography, proved essential both for navigation and complication management. While transaortic access remains a viable option, it should be approached with caution, necessitating a thorough understanding of device behaviour and a readiness to manage complications. These observations add important safety considerations for the expanded use of novel ablation platforms in patients with complex ventricular anatomy.

Learning points

- Large footprint catheter entrapment within the atrioventricular valve apparatus is a recognized risk, particularly when using a retrograde approach.
- Transseptal access should be preferred due to a lower risk of entrapment and the ability to facilitate catheter release (e.g. by advancing the sheath over the catheter).

## Introduction

Catheter ablation technology has evolved with the advent of integrated systems combining electroanatomic mapping and pulsed field ablation (PFA). Among these, the lattice-tip catheter (Sphere-9, Medtronic, Minneapolis, MN) is designed to deliver high-power radiofrequency (RF) and PFA through a single device, when used alongside a proprietary mapping system and generator (Affera™, Medtronic, Minneapolis, MN). Although this catheter is approved exclusively for atrial ablation, some features (i.e. large surface area and lattice structure) enable rapid anatomical reconstruction and targeted energy delivery, leading some centres to explore its off-label application in ventricular tachycardia (VT) ablation.

Initial experiences have shown promising results for VT treatment.^1–4^ However, safety data remain limited. Reported adverse events include coronary vasospasm, stroke or transient ischaemic attack, cardiac perforation, conduction system injury, phrenic nerve palsy, and valvular trauma.

To date, no report has addressed the interaction between this platform and the atrioventricular valvular apparatus. Here, we describe two cases of mechanical entrapment of the lattice-tip catheter during off-label VT ablation performed using a retrograde transaortic approach. In both instances, entrapment occurred within the subvalvular apparatus and was successfully resolved without lasting clinical consequences.

To our knowledge, these represent the first reported cases of such a complication, highlighting a previously unrecognized procedural risk. Caution is advised when navigating the lattice-tip catheter in the ventricles, especially while employing a retrograde approach.

## Summary figure

**Figure:**
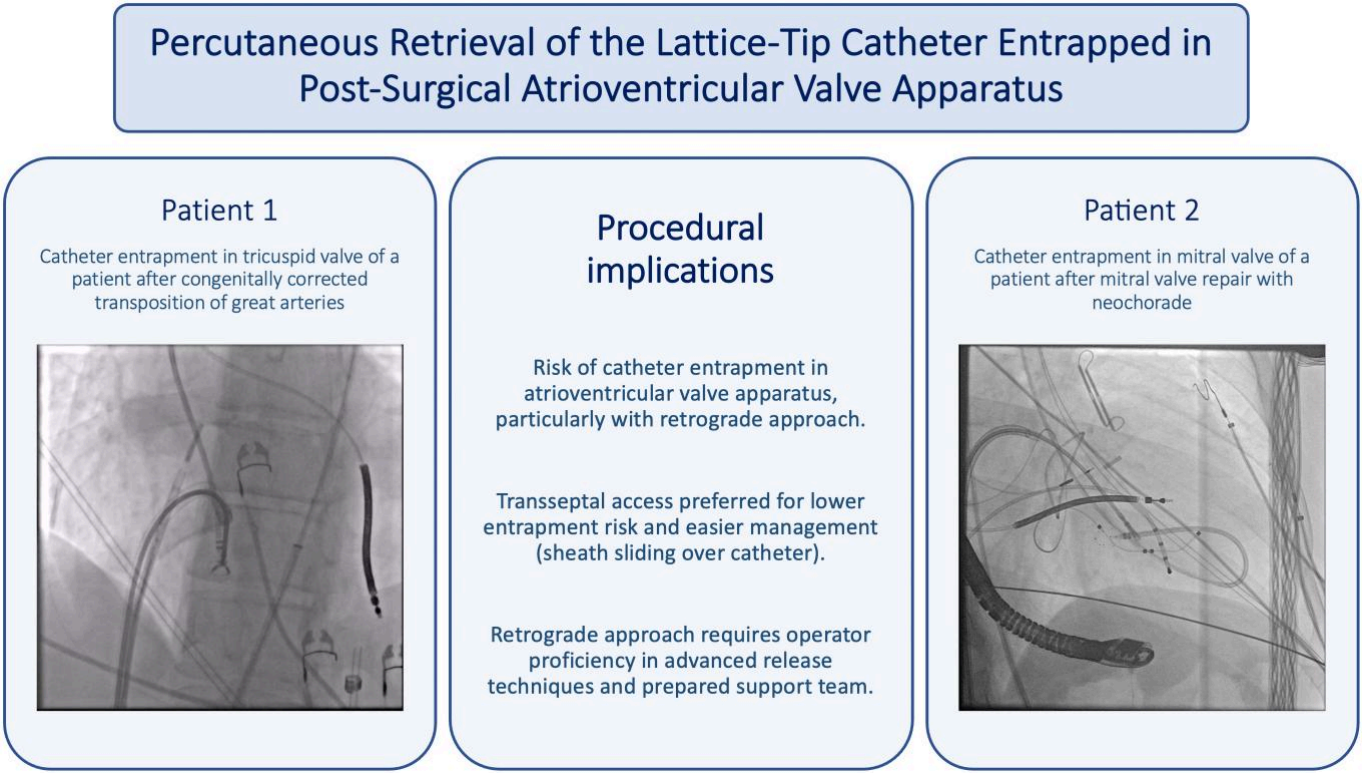


## Cases report

### Case 1

The first case involves a 38-year-old male with complex congenital heart disease who experienced catheter entrapment during a VT ablation procedure. He had a history of congenitally corrected transposition of the great arteries and a large ventricular septal defect (VSD), treated surgically with a Senning procedure, VSD closure using a Dacron patch, and ligation of the ductus arteriosus. In 2023, he survived an out-of-hospital cardiac arrest due to ventricular fibrillation (VF) and received a single-chamber implantable cardioverter-defibrillator (ICD), with the ventricular lead positioned in the morphological left ventricle (LV). In September 2024, while on bisoprolol 2.5 mg b.i.d., he developed recurrent monomorphic VT episodes terminated by antitachycardia pacing, prompting referral for catheter ablation. Transthoracic echocardiography showed dilated and dysfunctional systemic right ventricle (RV) with severe tricuspid regurgitation.

**Figure 1:**
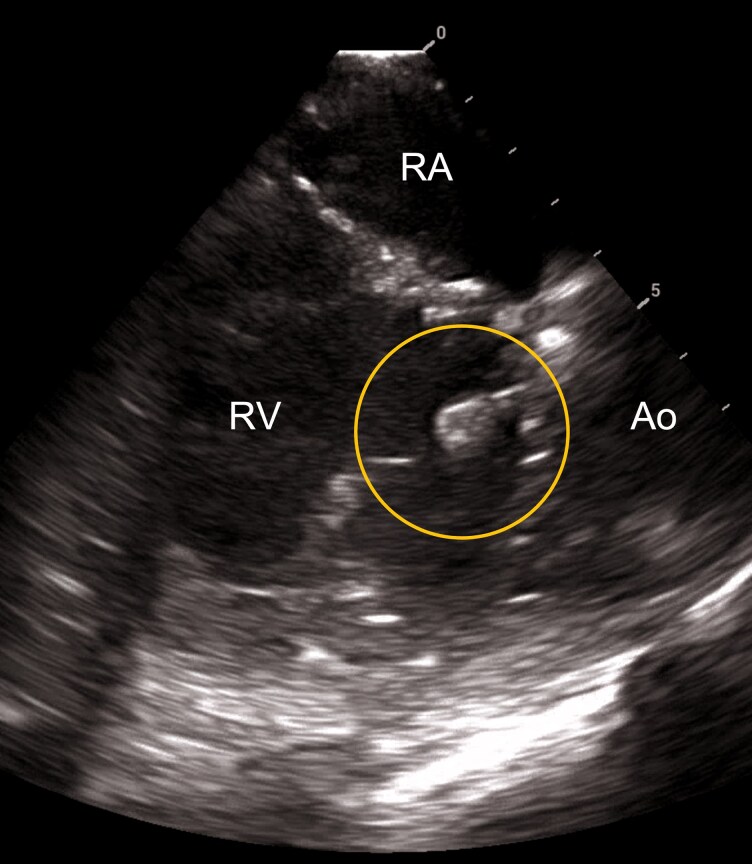
Catheter entrapment in the tricuspid valve apparatus during retrograde access. Intracardiac echocardiographic image illustrating the entrapment of the lattice-tip catheter (marked by the circle) within the tricuspid valve apparatus of a patient with congenitally corrected transposition of the great arteries following a Senning procedure. The systemic RV was accessed retrogradely via the aortic valve. Ao, aorta; RA, right atrium; RV, right ventricle.

**Figure 2:**
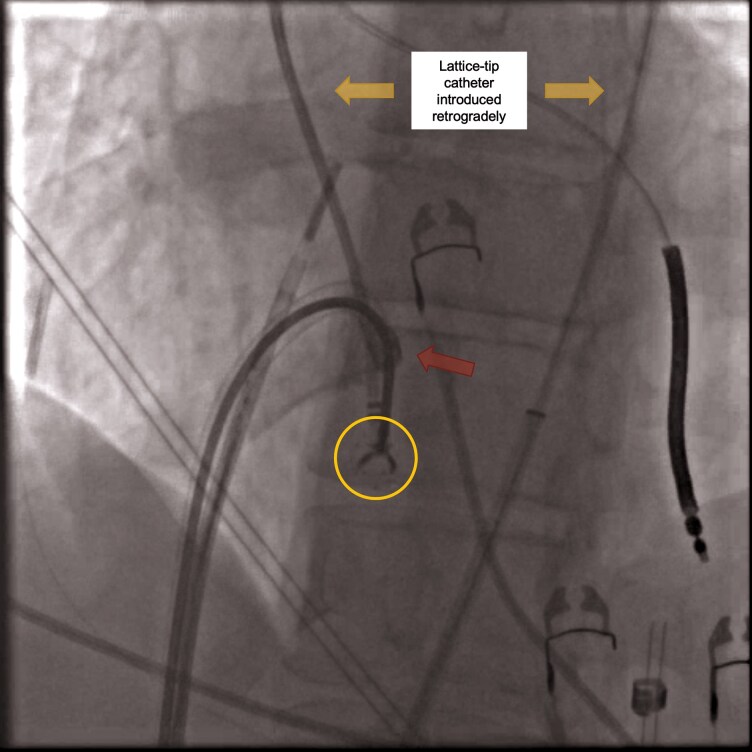
Catheter retrieval using grasping forceps via transbaffle puncture. Fluoroscopic image demonstrating the use of a transbaffle puncture to access the morphological right atrium. A grasping forceps (marked by the yellow circle) was advanced via a steerable sheath [(Faradrive, Boston Scientific) terminal part indicated by the red arrow] to grasp the distal portion of the lattice-tip ablation catheter (marked by the yellow arrows), which was entrapped in the subvalvular apparatus of the tricuspid valve.

**Figure 3:**
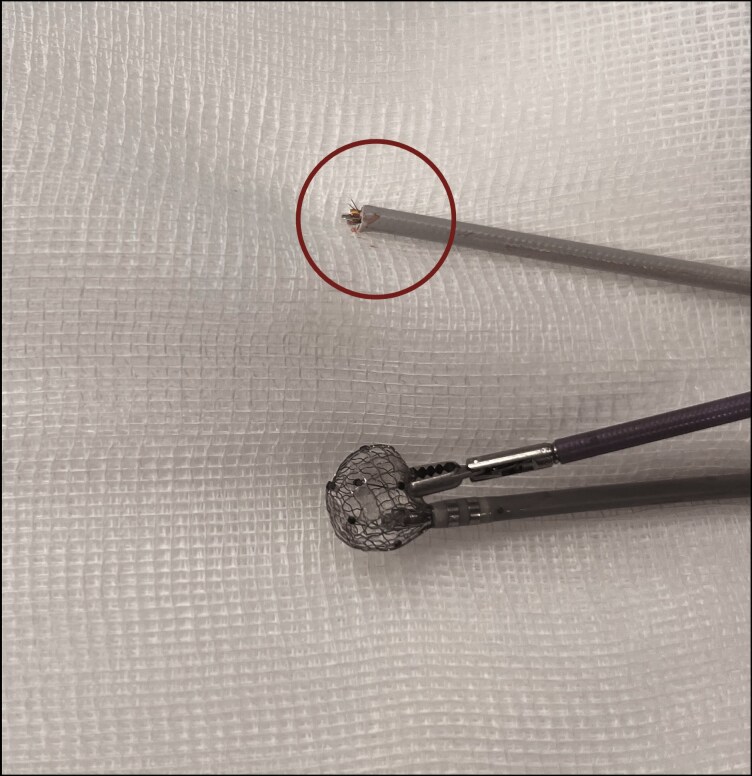
Full retrieval required to cut the proximal part of the lattice-tip catheter shaft. External view of the retrieval setup showing the proximal portion of the lattice-tip catheter (marked by the circle), which was cut to allow removal through the sheath. The image illustrates the relative dimensions between the grasping forceps and the deformed catheter tip.

Given the patient’s surgically altered anatomy, a retrograde transaortic approach was chosen to access the RV directly. The Sphere-9 catheter was selected due to the expected increased myocardial thickness in the systemic RV associated with congenital heart disease. Its dual mapping and ablation functions facilitated a seamless workflow without the need for catheter exchange. The procedure was performed under general anaesthesia with intracardiac echocardiographic (ICE) guidance. Initial programmed ventricular stimulation (PVS) from the LV induced only an unstable arrhythmia degenerating into VF, preventing the creation of an activation map. Therefore, the strategy shifted to a substrate-based approach, identifying dense scar as areas <0.5 mV and healthy myocardium as >1.5 mV. Additionally, sites of long (>40 ms) stimulus-QRS were systematically annotated. While manoeuvring the lattice-tip catheter retrogradely in the systemic RV, its distal portion became entrapped within the chordae tendineae of the tricuspid valve (*Figure 1*). Multiple traction attempts failed to release the catheter, raising concerns about potential valvular damage. Given that the patient had previously undergone a Senning procedure, the native interatrial septum had been surgically remodelled and largely replaced by a synthetic baffle, rendering a standard transseptal puncture anatomically impossible. For this reason, a transbaffle puncture represented the only feasible route to access the systemic venous atrium and was therefore performed. A Faradrive sheath (Boston Scientific) was advanced through the right femoral vein into the right atrium, and a Rat Tooth Grasping Forceps (Diversatek Healthcare, Milwaukee, WI)—typically used for gastrointestinal foreign bodies retrieval—was used under ICE guidance to grasp the catheter tip (*Figure 2*; Supplementary material online, *Video S1*), allowing its careful withdrawal into the sheath (see Supplementary material online, *Video S2*). To enable complete extraction, the proximal portion of the catheter was transected outside the patient’s body (*Figure 3*), permitting the removal of the entire assembly through the sheath.

**Figure 4:**
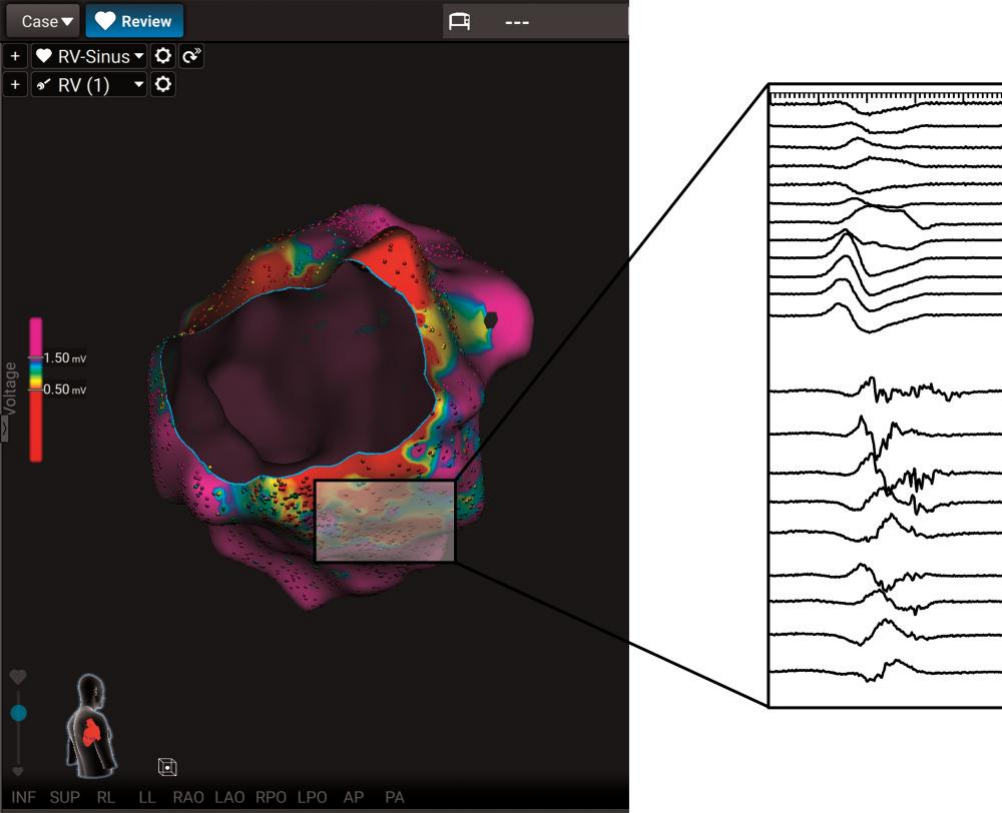
Substrate map of the systemic right ventricle (posterior–anterior view) showing an inferolateral scar with targeted local abnormal ventricular activities. Posterior–anterior view of the systemic right ventricle substrate map reveals a prominent inferolateral scar area characterized by low-voltage signals. The zoomed inset highlights local abnormal ventricular activities identified within the scar, which were specifically targeted during catheter ablation to modify the arrhythmogenic substrate. LAVAs, local abnormal ventricular activities.

A new lattice-tip catheter was then introduced through the transbaffle access, and a substrate map revealed an extensive inferolateral scar with multiple local abnormal ventricular activities (*Figure 4*). Pulsed field substrate modification along the tricuspid annulus was performed. A repeat PVS demonstrated no inducible arrhythmias. Post-procedural echocardiography confirmed the absence of pericardial effusion or worsening of tricuspid regurgitation.

The patient recovered uneventfully, remaining asymptomatic and free from VT recurrences at 3-month follow-up.

### Case 2

The second case involves a VT ablation procedure on a 60-year-old male with non-ischaemic dilated cardiomyopathy and previous mitral valve repair.

The patient received a dual-chamber pacemaker (His-bundle pacing) for Mobitz Type II atrioventricular block, later upgraded to a dual-chamber ICD after a VT episode, following inconclusive coronary angiography and cardiac magnetic resonance. Subsequent appropriate therapies for recurrent VT prompted a first endocardial ablation. During device interrogation, atrial fibrillation (AF) was also detected. The patient later experienced an appropriate shock for VF that caused syncope and a traumatic fall, complicated by subdural haematoma. This incident led to surgical closure of the left atrial appendage using an AtriClip Pro II (45 mm). Routine follow-up revealed a reduced LV ejection fraction (LVEF 35%), consistent with non-ischaemic dilated cardiomyopathy. Hence, the system was upgraded to a cardiac resynchronization therapy device for defibrillation (CRT-D) using a multifocal ventricular pacing configuration, accomplished by redirecting the His-bundle lead to the atrial port. Concomitant extraction of the atrial lead was performed. Subsequently, the patient underwent mitral valve repair for leaflet prolapse and annular dilation secondary to LV remodelling. Later, device interrogation revealed multiple episodes of tolerated VT, leading to the initiation of oral mexiletine and the scheduling of a second procedure.

**Figure 5:**
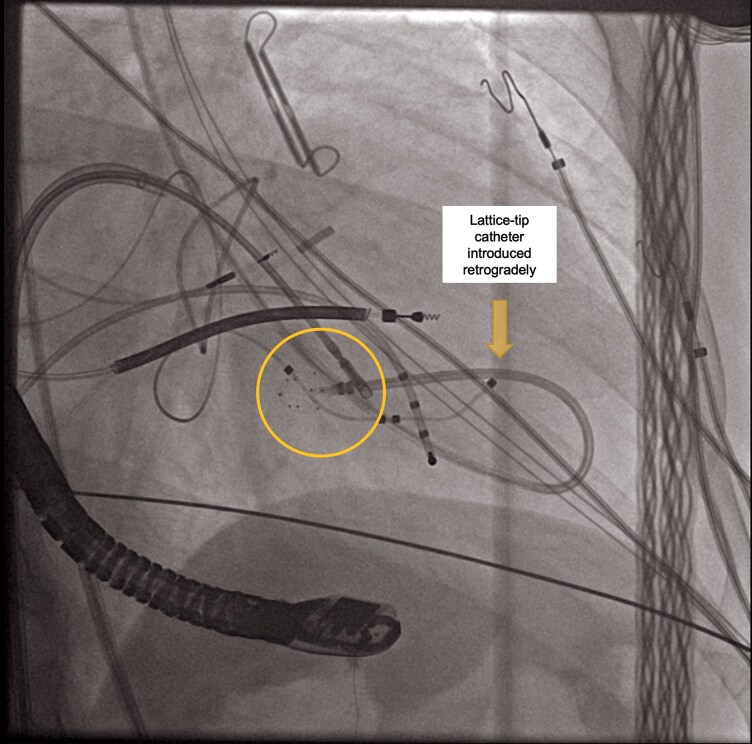
Fluoroscopic image (right anterior oblique view) depicting retrieval of the lattice-tip catheter entrapped within the neochordal apparatus. A bioptome, inserted through a steerable sheath, is advanced towards the lattice-tip catheter, which is caught between the chordae tendineae and the posteromedial papillary muscle. The bioptome jaws (highlighted by the circle) grasp the lattice-tip catheter to facilitate controlled extraction after unsuccessful traction attempts under echocardiography guidance.

Pre-procedural cardiac computed tomography showed basal-to-mid-antero-septal delayed enhancement with a mid-myocardial non-ischaemic pattern. Given the redo setting and the substrate location, ablation was planned using the lattice-tip catheter via a retrograde transaortic approach. The procedure was performed under general anaesthesia with ICE guidance. Programmed ventricular stimulation from the RV apex failed to induce sustained VT; hence, a substrate-based ablation strategy was pursued. Substrate mapping revealed low-voltage areas (<1.5 mV) in the anterior septum with late and fractionated potentials, along with dense scar (<0.5 mV) in the basal segments. During mapping, the lattice-tip catheter became entrapped between chordae tendineae and the posteromedial papillary muscle. Despite multiple ICE-guided attempts at gentle traction, the catheter could not be disengaged without risking excessive tension on the mitral apparatus. To enhance visualization of the interaction between the catheter and the subvalvular structures, transoesophageal echocardiography was introduced. A transseptal puncture was then performed, and an Agilis sheath (Abbott, St. Paul, MN) was advanced into the left atrium. Through the sheath, a solid-tip catheter was initially used to apply gentle counter-pressure at the shaft of the entrapped catheter, enabling partial mobilization. Subsequently, a bioptome was advanced via the same sheath and directed towards the distal portion of the lattice-tip catheter under combined transesophageal echocardiography and ICE guidance (*Figure 5*). Using controlled grasping and subtle rotational movements, the bioptome secured the catheter tip, allowing its safe disengagement from the chordae without imposing traction on the mitral leaflets. Continuous echocardiographic monitoring confirmed no disruption of the mitral apparatus or pericardial effusion. The procedure was completed using an irrigated-tip catheter (QDOT Micro, Biosense Webster) and the CARTO mapping system via the retrograde approach. Contiguous RF lesions (40–45 W) were delivered to homogenize the basal septal substrate. Final PVS was non-inducible for sustained VT. Follow-up transthoracic echocardiography showed stable mitral valve function.

## Discussion

To our knowledge, this is the first report documenting large-footprint catheter entrapment within the atrioventricular valvular apparatus.

While catheter entrapment is a recognized procedural complication, it remains relatively underreported in the literature. The earliest case was documented in 1994, when Conti *et al*.^5^ described the entrapment of an ablation catheter within the mitral valve during retrograde left accessory pathway ablation. Since then, though infrequently, various devices—including diagnostic catheters, ablation catheters,^6^ and guidewires^7^—have been implicated in similar occurrences. Entrapment within the tricuspid valve is uncommon, with the only existing report regarding a circular catheter, successfully managed using a double-snare technique.^8^

In a retrospective series of 348 AF ablation patients, Kesek *et al*.^9^ reported a 0.9% incidence of mitral valve entrapment with significant structural damage. Similarly, Cappato *et al*.^10^ documented non-specific valvular injuries in 0.07% of AF ablation procedures. Though relatively rare, these events are clinically significant and potentially life-threatening, since they carry a high risk of morbidity, necessitating conventional or minimally invasive surgical intervention in over half of cases.^6,9,11–13^ Even among patients managed percutaneously, ∼50% experience serious consequences, such as embolization of fractured catheter fragments or the need for subsequent mitral valve surgery due to iatrogenic damage.^6^

Most published cases involve circular catheters, which appear more prone to ensnare subvalvular chordae due to their helical configuration and protruding distal components, particularly when rotated counterclockwise.^6,14^ Circular catheters have also been identified as the most frequent cause of mitral valve injury during electrophysiological procedures.^11^ Conversely, reports involving ablation or multipolar catheters are rare, typically occurring in patients with mechanical prosthetic valves.^6^

It may be speculated that, compared to a conventional solid-tip catheter, the lattice-tip catheter carries a higher risk of entrapment, due to its larger and complex structure made of nitinol mesh. As the lattice tip is self-expanding, the distal tip can be collapsed only by withdrawal into the sheath. In the event of entrapment, transseptal access permits percutaneous rescue manoeuvres, which are typically not feasible via retrograde route, such as sheath advancement over the catheter shaft,^9^ or direct mechanical dislodgement by advancing the catheter towards the apical portion of the papillary muscle.^15^ For example, Mehta *et al*.^16^ reported resolution of a similar complication by grasping the entrapped catheter via a transseptally introduced snare, allowing withdrawal into the right atrium and ultimately the femoral vein—a method similar to that utilized in these two cases. Alternative methods—including diastolic prolongation with adenosine or rapid ventricular pacing—may also facilitate catheter disengagement through gentle traction.^17,18^

Based on our experience, the transseptal approach with large-footprint catheters should be favoured whenever feasible, as it appears to confer a superior safety profile in the context of VT ablation. Although transaortic access remains a viable option, it should be performed by experienced operators in settings equipped with surgical backup and advanced imaging modalities.

Real-time imaging is essential throughout the procedure. Both fluoroscopy and ICE play crucial roles in ensuring safe catheter navigation and facilitating prompt recognition of improper positioning. When using fluoroscopic guidance, right anterior oblique projection can help confirm the position of the catheter relative to the atrioventricular plane, utilizing reference structures such as a coronary sinus catheter.

Several practical recommendations may arise from our observations. First, if any resistance is encountered during catheter manipulation, movement should stop immediately. Forceful manoeuvres can lead to valvular trauma, necessitating surgical repair. Second, a prompt imaging assessment—preferably using intracardiac or transoesophageal echocardiography—is essential to evaluate the integrity of the valve apparatus at the time of entrapment and after retrieval to rule out iatrogenic valvular damage.^19^ Finally, any retrieved hardware should be carefully inspected to ensure its integrity and confirm that no components have been retained within the cardiovascular system. This step is crucial to reduce the risk of delayed embolic complications.

This report is limited by its descriptive nature and small sample size. Further studies are needed to assess the incidence, contributing factors, and optimal prevention strategies for lattice-tip catheter entrapment in atrioventricular valves.

## Conclusion

These cases highlight a previously unrecognized risk of entrapment in the atrioventricular valves when using the lattice-tip catheter for VT ablation, particularly when choosing a retrograde transaortic approach. The combination of the catheter’s large footprint and complex subvalvular anatomy predisposes to entanglement, potentially leading to valvular damage. Therefore, transseptal access should be preferred whenever possible, as it provides a more favourable trajectory and enables safer and more effective percutaneous bailout strategies. Intracardiac echocardiography is essential for real-time monitoring during catheter manipulation near the valvular apparatus and for complication management. While transaortic access is not absolutely contraindicated, its use demands a clear understanding of the associated limitations and mechanical risks, along with adequate preparation for advanced percutaneous rescue interventions.

## Lead author biography


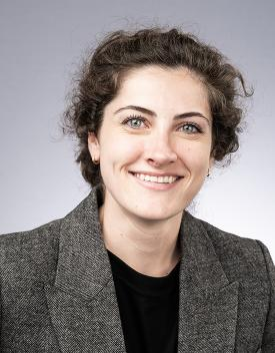


Nicoletta Ventrella, MD, is a cardiologist with a special interest in cardiac electrophysiology at the Institute of Clinical and Experimental Medicine in Prague, Czech Republic. She obtained her medical degree from Università Campus Bio-Medico of Rome, Italy, in 2020, and subsequently completed her residency in cardiology at the University of Milan, in 2025. Her current clinical and research work focuses on the invasive management of atrial and ventricular arrhythmias. Dr Ventrella is dedicated to advancing knowledge in cardiac electrophysiology through both clinical practice and scientific research.

## Supplementary Material

ytag077_Supplementary_Data

## Data Availability

The data underlying this article cannot be shared publicly due to the privacy of individuals that participated in the study. The data will be shared on reasonable request to the corresponding author.
